# Assessment of Thrombin Generation in Pediatric Patients on Extracorporeal Life Support: Impact of Bivalirudin and Other Anticoagulants

**DOI:** 10.1111/ijlh.70223

**Published:** 2026-08-16

**Authors:** Vadim Kostousov, Karen Bruzdoski, Amir Navaei, Jun Teruya

**Affiliations:** ^1^ Department of Pathology & Immunology Baylor College of Medicine Houston Texas USA; ^2^ Department of Pathology, Division of Transfusion Medicine & Coagulation Texas Children's Hospital Houston Texas USA; ^3^ Department of Pediatrics, Division of Critical Care Medicine & Cardiology Baylor College of Medicine Houston Texas USA; ^4^ Department of Medicine Baylor College of Medicine Houston Texas USA

**Keywords:** bivalirudin, enoxaparin, heparin, thrombin generation, warfarin

## Abstract

**Introduction:**

Bivalirudin is an alternative anticoagulant to heparin and is widely used in pediatric extracorporeal life support (ECLS). Its activity is monitored using activated partial thromboplastin time (aPTT) and/or more specific tests such as plasma‐diluted thrombin time (dTT). A new automated thrombin generation assay (TGA) has also shown promise in evaluating the hemostatic response in anticoagulated patients; however, its utility for monitoring bivalirudin or other anticoagulants in pediatrics remains unknown. We aimed to evaluate TGA in pediatric patients treated with bivalirudin and to compare it with that observed in patients receiving other commonly used anticoagulants.

**Methods:**

TGA, aPTT, aPTT with hepzyme, dTT, INR were measured in 30 specimens from 12 pediatric ECLS patients anticoagulated with bivalirudin. In addition, TGA, anti‐Xa assay, and INR were measured in samples from children receiving enoxaparin (*n* = 22, 10 patients), heparin (*n* = 16, 8 patients), and warfarin (*n* = 19, 6 patients).

**Results:**

TGA lag ratio showed a strong correlation with bivalirudin concentration as measured by dTT and ROC analysis demonstrated that a Lag ratio > 2.0 predicted specimens within the therapeutic range for bivalirudin (sensitivity 0.88, specificity 0.67, accuracy 0.79). Interestingly, samples containing endogenous heparin‐like substances exhibited paradoxically enhanced thrombin generation, despite having the highest aPTT values. Enoxaparin effect was associated with a reduction in thrombin peak and endogenous thrombin potential (ETP) suppression, while both heparin and warfarin led to a prolonged lag time, along with decreased peak and greater ETP suppression.

**Conclusion:**

TGA shows promise in evaluating bivalirudin anticoagulation and is capable of detecting anticoagulant activity across both subtherapeutic and therapeutic levels of other agents commonly used in pediatric patients.

## 1 Introduction

Bivalirudin is an effective alternative to unfractionated heparin (hereafter referred to as heparin) for anticoagulation in pediatric extracorporeal life support (ECLS) and may be associated with improved safety outcomes [1]. The anticoagulant effect is usually monitored by activated partial thromboplastin time (aPTT); at our institution, aPTT with heparinase (HPTT) was used for many years to monitor anticoagulation, with a target range of 60–80 s prior to the implementation of direct bivalirudin measurement using plasma‐diluted thrombin time (dTT).

The thrombin generation assay (TGA) is utilized to evaluate coagulation cascade response, initiated by tissue factor. It can be used to detect coagulation abnormalities or prothrombotic state, as well as monitoring a response to various anticoagulants [2]. Automation and concurrent measurement of normal plasma control sample in TGA enable standardization of results and support its potential for clinical application.

The main purpose of our study is to evaluate thrombin generation in pediatric patients on ECLS treated with bivalirudin and to compare with other coagulation tests. Additionally, we aim to examine the bivalirudin‐induced changes in TGA and compare them with those observed with other anticoagulants commonly used in pediatric patients.

## 2 Methods

After Institutional Review Board approval, 30 samples from 12 pediatric patients receiving bivalirudin therapy during extracorporeal membrane oxygenation (ECMO) or ventricular assist device (VAD) support were collected and analyzed. Testing included activated partial thromboplastin time (aPTT, STA‐PTT Automate, Diagnostica Stago), heparinase‐modified (heparinase I > 125 U/mL) partial thromboplastin time (HPTT, Dade Hepzyme, Siemens Healthcare Diagnostics, Newark, Delaware, USA), prothrombin time (STA‐NeoPTimal, ISI ~ 1, Diagnostica Stago), anti‐Xa assay (STA‐Liquid Anti‐Xa, Diagnostica Stago) and bivalirudin assay using the STAR Max analyzer (Diagnostica Stago, Parsippany, NJ, USA). Thrombin generation assay (TGA) was performed on the ST Genesia analyzer (Diagnostica Stago, Parsippany, NJ, USA) using the STG DrugScreen reagent. Although the exact composition of the STG DrugScreen reagent is proprietary, it is reported to be comparable to the “CAT PPP‐Reagent High”, which includes 20 pM tissue factor and 4 μM phospholipids [3]. To minimize the risk of heparin contamination, specimens were collected either via peripheral venipuncture or through lines flushed with a nonheparin solution. When specimens were collected from an indwelling intravenous line containing a heparin flush, the first 10 mL of blood was discarded to reduce contamination risk. Specimen selection was limited to those with presumably therapeutic aPTT values (> 60 s) and was not collected at the time of anticoagulation initiation or ECMO circuit change. Specimens are considered to contain heparin‐like substances (HLS) if the aPTT/HPTT ratio > 1.1, as suggested earlier [4]. Bivalirudin concentration was measured with 1:10 dTT (STA‐Thrombin, Diagnostica Stago) as described before [5]. Briefly, one volume of patient citrated plasma was mixed with nine volumes of normal pooled plasma (NPP, Precision Biologics, Dartmouth, NS, Canada). Then, 100 μL of this diluted plasma was added to 100 μL of STA thrombin reagent, and the clotting time was recorded. Using a standard curve generated with bivalirudin calibrators, the assay converts the observed clotting time into the corresponding drug concentration in plasma. The assay was fully automated, with a target bivalirudin range of 0.7–2.2 μg/mL, corresponding to an aPTT of 60–80 s. This range was established using samples spiked with varying concentrations of bivalirudin (Figure S1). Absolute values of TGA variables (Lag, min; Thrombin Peak, nmol/L; endogenous thrombin potential (ETP), nmol/L × min) were normalized as Lag ratio, Peak %, ETP % by reference plasma values. Additionally, specimens were collected for TGA analysis from children receiving enoxaparin (*n* = 22, 10 patients), heparin (*n* = 16, 8 patients), and warfarin (*n* = 19, 6 patients). International normalized ratio (INR) and anti‐Xa activity values were extracted from the database. Target range was defined as INR 2.0–3.0 for warfarin and anti‐Xa 0.3–0.6 IU/mL for heparin. For enoxaparin a cutoff ≥ 0.6 IU/mL was applied due to lack of specimens with subtherapeutic level (< 0.45 IU/mL). Statistical analyses, including the Mann–Whitney U test, Receiver Operating Characteristic (ROC) curve analysis and Pearson correlation (*r*), were performed using Microsoft Excel version 14.0 (2010; Redmond, WA, USA). Data are presented as mean ± SD, and significance was set at *p* < 0.05. Correlations were categorized as follows: 0.20–0.39 as weak, 0.40–0.59 as moderate, 0.60–0.79 as strong, and 0.80–1.00 as very strong.

## 3 Results

Patient demographics are presented in Table 1. Among 30 specimens from patients receiving bivalirudin, the mean aPTT was 80.2 ± 15.2 s and HPTT 68.2 ± 8.0 s, bivalirudin level was 0.88 ± 0.48 μg/mL and INR 2.1 ± 0.6. TGA lag phase averaged 2.88 ± 1.44 min, representing a 2.6 ± 1.3‐fold prolongation compared to reference plasma (RP). The absolute peak thrombin concentration was 356 ± 49 nmol/L (73.5% ± 9.6% of RP), and the endogenous thrombin potential (ETP) was 1222 ± 229 nmol/L × min (69.4% ± 11.6% of RP) (Figure 1). Specimens were categorized based on bivalirudin levels as either subtherapeutic (< 0.7 μg/mL) or within the target therapeutic range (TTR = 0.7–2.2 μg/mL). Thirteen samples fell into the subtherapeutic range (0.24–0.65 μg/mL), while 17 were within the TTR (0.71–2.16 μg/mL) (Table 2).

**TABLE 1:** Patient demographics.

Anticoagulant	Gender (M/F)	Age, years (median, IQR)	Indications
Bivalirudin (12 patients, 30 specimens)	8/4	1.3 (0.3–17)	ECMO 7, VAD 5
Heparin (8 pts., 16 specimens) Enoxaparin (10 pts., 22 specimens) Warfarin (6 pts., 19 specimens)	15/9	2 (0.6–17)	ECMO 7, VAD 5, other indications (mostly thrombosis) 12

Abbreviations: ECMO—extracorporeal membrane oxygenation; IQR—interquartile range; M/F—male/female; VAD—ventricular assist device.

**FIGURE 1:**
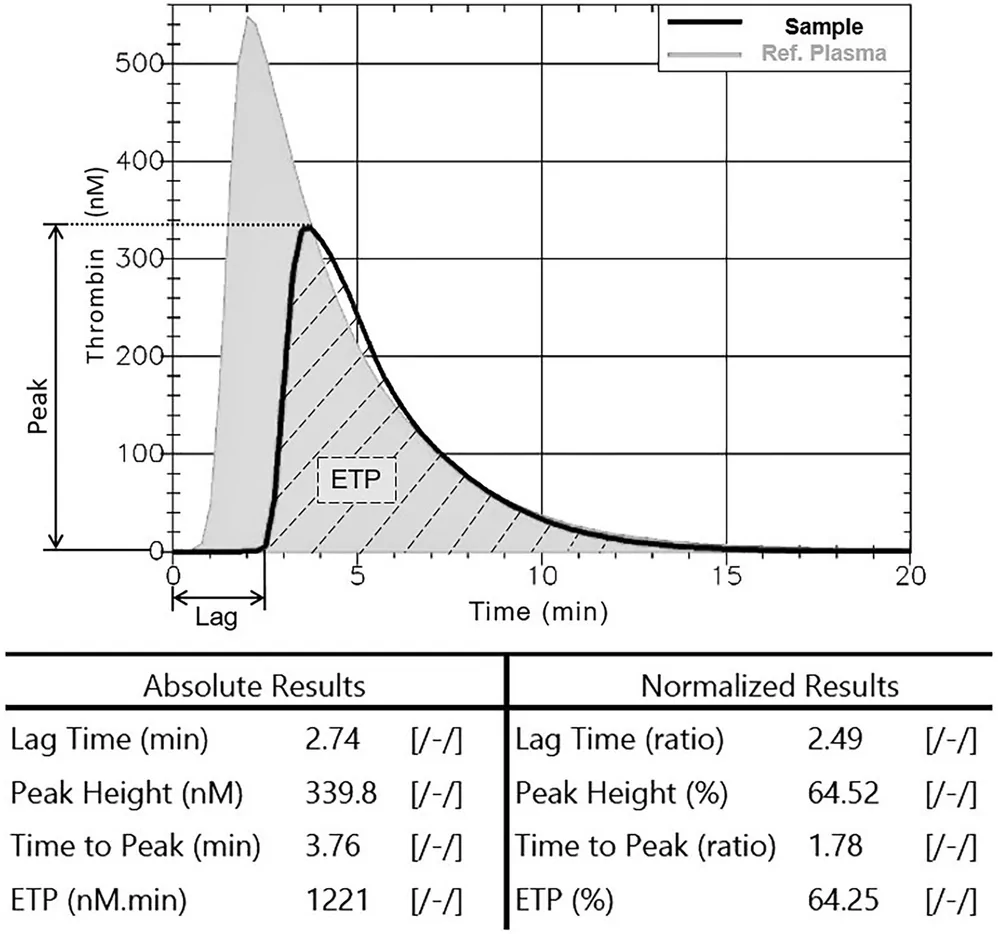
The representative tracing of thrombin generation in normal reference (gray shadow) and bivalirudin‐treated plasma (sample), patient on VAD, bivalirudin concentration 1.15 μg/mL, aPTT 99.7 s, HPTT 73.7 s, INR 2.4. ETP—endogenous thrombin potential (area under the curve).

**TABLE 2:** Coagulation tests results in samples anticoagulated with bivalirudin.

Test variable	Bivalirudin, μg/mL	aPTT, sec	HPTT, sec	INR	Lag ratio	Peak ratio, %	ETP ratio, %
Below TR (*n* = 13, 6 pts)	0.46 ± 0.12	77.5 ± 16.8	65.6 ± 7.0	1.9 ± 0.4	1.8 ± 0.4	78.1 ± 8.1	69.1 ± 9.8
On TR (*n* = 17, 9 pts)	1.20 ± 0.38	82.4 ± 14.0	70.2 ± 8.3	2.4 ± 0.6^a^	3.2 ± 1.4^a^	70.0 ± 7.8^a^	69.7 ± 13.7

^a^ *p* < 0.05 compared to values below therapeutic range (TR).

Lag ratio was significantly prolonged, while both absolute peak values and peak ratios were reduced in specimens within the TTR. Interestingly, higher INR values were also predictive of specimens within the TTR. Strong correlations were observed between bivalirudin levels and both Lag time (*r* = 0.68, *p* < 0.0001) and INR (*r* = 0.71, *p* < 0.0001). ROC analysis showed that a lag ratio > 2.0 predicted specimens within the TTR (AUC = 0.86, *p* < 0.0001; sensitivity 0.88; specificity 0.67; accuracy 0.79), whereas an INR ≥ 2.1 predicted a therapeutic bivalirudin level with a sensitivity of 0.71, specificity of 0.83, and accuracy of 0.76 (AUC = 0.80, *p* < 0.001). Among other variables only peak ratio showed a moderate correlation (*r* = −0.52, *p* = 0.004). ROC analysis indicated that a peak ratio of < 72% was predictive of a specimen within the TTR (AUC = 0.82, *p* = 0.223; sensitivity 0.71; specificity 0.91; accuracy 0.79). HLS were detected in 63% of the analyzed specimens. Among these, only aPTT was significantly higher in HLS positive specimens (84.7 ± 17.0 vs. 72.5 ± 6.8 s, *p* = 0.031). Interestingly, TGA in specimens with HLS revealed paradoxical enhanced thrombin generation characterized by a nonsignificantly shorter lag ratio (2.5 ± 1.1 vs. 2.7 ± 1.5, *p* = 0.59) and significantly higher peak ratio (76.7 ± 8.9 vs. 67.8 ± 8.1, *p* = 0.010) compared to specimens without HLS.

Other anticoagulants also demonstrated a dose‐dependent pattern of TGA inhibition. Enoxaparin was associated with a reduction in thrombin peak and enhanced ETP suppression. In contrast, both heparin and warfarin led to a prolonged lag time, along with decreased peak and greater ETP suppression. Two specimens from patients on warfarin with INR values of 3.5 and 3.7 exhibited unmeasurable thrombin generation. All affected TGA variables showed significant differences when comparing therapeutic versus subtherapeutic values of routine monitoring assays, as well as between low and higher therapeutic anti‐Xa activity levels in patients receiving enoxaparin (Table 3). Additionally, significant strong correlations were found for INR versus lag ratio (*r* = 0.82, *p* < 0.001), peak (*r* = −0.89, *p* < 0.001), and ETP (*r* = −0.77, p < 0.001); anti‐Xa activity for enoxaparin versus peak (*r* = −0.61, *p* = 0.002), and ETP (*r* = −0.62, *p* = 0.002). Anti‐Xa activity for heparin correlated moderately with lag ratio (*r* = 0.48, *p* = 0.06), peak (*r* = −0.49, *p* = 0.054), ETP (*r* = −0.47, *p* = 0.07).

**TABLE 3:** TGA variables in specimens treated with other anticoagulants.

Drug	Range	Test values	Lag ratio	Peak, %	ETP, %
Enoxaparin (10 patients)	Anti‐Xa < 0.6 (*n* = 11)	Anti‐Xa 0.46 ± 0.06 IU/mL	1.5 ± 0.3	57.9 ± 20.2	51.7 ± 14.1
	Anti‐Xa ≥ 0.6 (*n* = 11)	Anti‐Xa 0.77 ± 0.2 IU/mL	1.6 ± 0.5	30.0 ± 16.0^a^	31.3 ± 11.1^a^
Heparin (8 patients)	Below TR (*n* = 7)	Anti‐Xa 0.22 ± 0.05 IU/mL	1.1 ± 0.1	48.9 ± 15.1	33.4 ± 10.3
	On TR (*n* = 9)	Anti‐Xa 0.44 ± 0.1 IU/mL	1.3 ± 0.2^a^	25.0 ± 14.3^a^	22.9 ± 6.2^a^
Warfarin (6 patients)	Below TR (*n* = 6)	INR 1.6 ± 0.2	1.4 ± 0.3	43.6 ± 8.6	43.5 ± 11.4
	On TR (*n* = 11)	INR 2.6 ± 0.2	2.3 ± 0.4^a^	24.1 ± 7.3^a^	24.5 ± 6.3^a^

^a^ *p* < 0.05 compared to values below TR (therapeutic range).

## 4 Discussion

To the best of our knowledge, this is the first study to evaluate new ST Genesia DrugScreen TGA in plasma samples from an anticoagulated pediatric population. The ST Genesia Drugscreen results correlated variously with the concentrations or activity of the drug measured by established routine coagulation assays. A recent publication investigating the impact of bivalirudin on thrombin generation in pediatric plasma employed the Calibrated Automated Thrombogram (CAT) assay, using pooled specimens spiked with varying bivalirudin concentrations [6]. Our findings align with this study, indicating a consistent increase in the lag time across different age groups when bivalirudin concentration transitioned from 0.5 to 1.0 μg/mL. When examining the responses, both peak and ETP demonstrated a paradoxical increase within this range. In contrast, our analysis of individual patient specimens revealed a mild but statistically significant decrease in peak, while ETP remained unchanged when comparing specimens with a mean bivalirudin concentration of 0.5 μg/mL to those with 1.2 μg/mL in plasma. Differences in assay performance or reagent formulations (5 pM of tissue factor (TF) in CAT vs. 20 pM TF for ST DrugScreen, possible difference in phospholipid composition) could also be responsible for these discrepant findings. The bivalirudin reference range used at our institution is consistent with previously published data (as summarized in Table 6 of Reference 1) and is largely determined by the historical aPTT therapeutic targets and the sensitivity of the aPTT reagents to bivalirudin. This range varies from 0.5 to 2.5 μg/mL, corresponding to an aPTT ratio of 1.5 to 2.5 times baseline. Our observation of an INR correlation to bivalirudin levels is supported by previous findings, where the INR correlated more strongly with the bivalirudin infusion rate (*r* ^2^ = 0.683) than the aPTT (*r* ^2^ = 0.476) in a pediatric ECMO setting [7]. Another study demonstrated that the highest correlation between INR and aPTT in pediatric paracorporeal VAD patients occurred only after heparin contamination from the sampling line was neutralized [8]. Heparin‐like substances, which are endogenous glycosaminoglycans—including heparan sulfate, chondroitin sulfate, and dermatan sulfate—exhibit a heparin‐like effect by prolonging coagulation tests. These substances have been described in ECMO patients anticoagulated with bivalirudin [9]. Previously we reported that about 50% of pediatric specimens on ECLS had HLS and we demonstrated that HLS affect negatively monitoring of bivalirudin by aPTT [5]. We have now demonstrated that the presence of HLS in plasma is associated with enhanced thrombin generation, suggesting that HLS may contribute to a prothrombotic profile, particularly in subtherapeutic bivalirudin conditions. Circulating microparticles in specimens with HLS may contribute to augmented thrombin generation, as previously demonstrated for platelet‐derived microparticles and elevated thrombin peak values in patients with thrombosis [10]. Furthermore, endogenous heparinization was identified in severely traumatized patients via thromboelastography (comparing kaolin‐activated and kaolin‐heparinase–activated specimens). This was accompanied by increased circulating thrombomodulin and syndecan‐1, indicating degradation of the endothelial glycocalyx and profound endothelial damage [11]. Recent studies have reported variable TGA responses measured with the ST Genesia analyzer in adults treated with direct oral anticoagulants [12] and other anticoagulants [13]. Helin et al. reported findings closely aligned with ours, demonstrating similar thrombin generation profiles in adult pooled plasma anticoagulated with warfarin at an INR of 2.5 [13]. They also observed undetectable thrombin generation in specimens with INR > 3.0, consistent with our observations in pediatric specimens [13]. However, at the same level of anti‐Xa 0.5 IU/mL in pooled adult plasma from patients treated with low molecular weight heparins (LMWH), pediatric specimens exhibited a shorter lag time and higher peak and ETP values. This discrepancy may be attributed to differences in LMWH utilization, age‐dependent LMWH pharmacokinetics, or variations in anti‐Xa assay methodology. Notably, the published observation [13] employed an anti‐Xa assay containing dextran sulfate, which is known to mobilize LMWH bound to plasma proteins, thereby increasing measured anti‐Xa activity in the sample. Another study in adults receiving heparin and low molecular weight heparin (enoxaparin) found that, within the therapeutic heparin subgroup, lag time showed the strongest correlation with anti‐Xa levels [14]. In the therapeutic LMWH subgroup, a significant correlation was observed between anti‐Xa activity and thrombin peak—findings that align closely with our own observations. Our study has several limitations. Notably, we did not adjust for age when comparing specimens from different patients, which means that some observed changes in TGA variables may be partially attributable to age‐related developmental differences [15] or underlying disease in pediatric patients. There may also be a degree of selection bias, as the requirement for a minimum specimen volume of 600 μL of residual citrated plasma limited the inclusion of samples suitable for TGA measurement on the ST Genesia analyzer.

## 5 Conclusion

We conclude that the ST Genesia DrugScreen assay demonstrates promising utility in evaluating the anticoagulant effect of bivalirudin on thrombin generation. Moreover, it effectively detects anticoagulant activity across both subtherapeutic and therapeutic levels of other agents currently used in pediatric patients. Further studies are warranted to determine whether this assay can provide additional insights or should be used in conjunction with traditional methods for monitoring anticoagulation in pediatric patients.

## Author Contributions

J.T. designed the study. K.B. and V.K. performed data acquisition and data analysis. V.K., A.N., and J.T. interpreted the results. V.K. made a significant contribution to the initial manuscript draft. V.K., K.B., A.N., and J.T. made critical revisions to the final paper. All authors critically reviewed the manuscript.

## Funding

The authors have nothing to report.

## Ethics Statement

Institutional Review Board approved this study without patient consent.

## Conflicts of Interest

The authors declare no conflicts of interest.

## Supporting information


**Figure S1:** To establish the bivalirudin therapeutic range, normal pooled plasma was spiked with bivalirudin at final concentrations of 0.125–2.5 μg/mL. aPTT measurements were performed in triplicate, and mean values were plotted as a function of bivalirudin concentration. The lower aPTT target of 60 s corresponded to 0.7 μg/mL, whereas the 80‐s target corresponded to 2.2 μg/mL.

## Data Availability

Author elects to not share data.
