# Supplementary material for: Assessment of Thrombin Generation in Pediatric Patients on Extracorporeal Life Support: Impact of Bivalirudin and Other Anticoagulants
Source: Int J Lab Hematol. 2026 Aug 16;48(6):1472–6. doi: 10.1111/ijlh.70223 (PMC13729925; doi:10.1111/ijlh.70223)
Supplement: Supplementary file 1 [file IJLH-48-1472-s001.zip › ijlh70223-sup-0001-FigureS1@Supplementary Figure 1 Legend.docx]

**Supplementary Figure 1.** To establish the bivalirudin therapeutic range, normal pooled plasma was spiked with bivalirudin at final concentrations of 0.125–2.5 µg/mL. aPTT measurements were performed in triplicate, and mean values were plotted as a function of bivalirudin concentration. The lower aPTT target of 60 seconds corresponded to 0.7 µg/mL, whereas the 80‑seconds target corresponded to 2.2 µg/mL.
